# Effects of a maternal–infant telecare program on postpartum maternal confidence and sleep quality of mothers and infants

**DOI:** 10.1038/s41598-026-41565-5

**Published:** 2026-02-28

**Authors:** Ching-Yi Lai, Wei-Sho Ho, Ko-Chien Liu, Hui-Ling Hsiao, Wan-Yun Hsu, Wei-Lun Huang

**Affiliations:** 1 Department of Nursing, Central Taiwan University of Science and Technology, No. 666, Buzi Rd., Beitun Dist, Taichung City, 406053 Taiwan; 2 Department of Electrical and Mechanical Technology, National Changhua University of Education, Bao-Shan Campus, No.2, Shi-Da Road, Changhua City, 500208 Taiwan; 3 Graduate Institute of Technological and Vocational Education, National Changhua University of Education, Bao-Shan Campus, No. 2, Shi-Da Road, Changhua City, 500208 Taiwan; 4 NCUE Alumni Association, National Changhua University of Education, Jin-De Campus, No. 1, Jinde Rd, Changhua City, 500207 Taiwan; 5 Infancix Postpartum Nursing Care Center, No. 32-3, Ln. 103, Sec. 1, Xinsheng S. Rd., Da’an Dist, Taipei City, 106084 Taiwan; 6 Baopu Development, 1 F., No. 90, Ln. 134, Sec. 3, Xinyi Rd., Da’an Dist, Taipei City, 106023 Taiwan; 7 College of Management, NTU-Fudan EMBA, National Taiwan University, No. 1, Sec. 4, Roosevelt Rd., Da’an Dist, Taipei City, 106319 Taiwan; 8 International Medical Service Center, Taichung Veterans General Hospital, No. 1650, Sec. 4, Taiwan Blvd., Xitun Dist, Taichung City, 407219 Taiwan; 9 Medical Affairs Office, National Taiwan University Hospital, No. 7, Zhongshan S. Rd., Zhongzheng Dist, Taipei City, 100225 Taiwan; 10 Department of Health Services Adminstration, China Medical University, No. 100, Sec. 1, Jingmao Rd., Beitun Dist, Taichung City, 406040 Taiwan; 11 Department of Health Care Management, National Taipei University of Nursing and Health Sciences, No. 365, Mingde Rd., Beitou Dist, Taipei City, 112303 Taiwan

**Keywords:** Maternal-infant telecare, Maternal confidence, Sleep quality, Health care, Medical research

## Abstract

The change in the medical payment system has shortened postpartum hospitalization, limiting the time for nursing staff to provide care and education. In Taiwan, many postpartum women choose “doing the month” in postpartum nursing care centers with professional support. However, leaving these centers often increases maternal stress as they transition to independent caregiving, impacting their confidence and sleep quality. Additionally, difficulties in establishing infants’ sleep patterns further disrupt maternal sleep. To address these challenges, the maternal-infant telecare program was introduced to provide continued support after discharge. To explore the effectiveness of the maternal-infant telecare program on postpartum women’s confidence, sleep quality, and infants’ sleep quality. A cross-sectional research design was used in this study. Eighty-two postpartum women were recruited from a postpartum nursing care center in Northern Taiwan. Data were gathered at three separate time points (pretest, Posttest 1, Posttest 2). All statistical tests were performed using the SPSS 24 software. Data analyses included descriptive statistics, independent t-tests, and Generalized Estimating Equation. A total of eighty-two postpartum women were recruited through convenience sampling. The results of this study showed that after the intervention, postpartum confidence increased from an average of 43.99 to 51.72, postpartum sleep quality improved from 23.35 to 19.33, and the number of infants’ nighttime awakenings, as reported by postpartum women, reduced from an average of 2.77 to 1.12 at twelve weeks. The maternal-infant telecare program demonstrated potential benefits in enhancing postpartum women’s confidence, improving sleep quality, and reducing the frequency of infants’ nighttime awakenings. Therefore, the program merits further promotion and broader application in postpartum care.

## Introduction

With advances in modern healthcare systems, postpartum hospital stays have significantly shortened, limiting the time available for healthcare professionals to provide postpartum care and health education. As a result, postpartum women face increasing challenges in managing care after discharge^1^. These women must not only focus on physical recovery but also learn essential newborn care skills—tasks that are particularly daunting for first-time mothers^2^. Moreover, the irregular sleep patterns of newborns frequently disrupt maternal sleep, and persistent sleep deprivation can heighten the risk of postpartum psychological and physical health issues^3^.

In traditional Chinese culture, postpartum women are considered physically weakened due to exertion, sweating, and significant blood loss during childbirth. Consequently, a culturally embedded practice known as “doing the month”is followed for one month to facilitate recovery^4^. Historically, this care was provided within the family setting, with relatives supporting the mother’s recuperation and helping her adapt to infant care demands. However, shifts in family structures and increasing care needs have led many postpartum women in Taiwan to opt for professional postpartum nursing care centers. These facilities offer comprehensive services including health monitoring, individualized nursing guidance, breastfeeding support, postpartum rehabilitation exercises, and dietary regimens based on traditional postpartum nutrition principles^5,6^. With professional support, mothers can better regain their strength and develop essential infant care skills, while newborns benefit from systematic monitoring and safe care.

Nonetheless, the real challenge often begins after discharge from these centers, when mothers return home and assume sole or family-supported responsibility for newborn care. Tasks such as frequent breastfeeding, diaper changes, and soothing routines can overwhelm inexperienced mothers, potentially undermining their maternal confidence and sleep quality^3,7,8^. Meanwhile, their own physical recovery and self-care must continue to ensure holistic postpartum well-being.

To address these post-discharge challenges, telecare has emerged as an effective solution. Utilizing digital platforms and communication technologies, telecare enables healthcare professionals to remotely monitor maternal and infant health and offer timely education and guidance. This includes support for physical recovery, breastfeeding, infant sleep regulation, and common concerns^9,10^. In response to the needs of mothers transitioning home from postpartum nursing care centers, this study developed a Maternal-Infant Telecare Program (MITP). The study aims to evaluate the program’s impact on maternal confidence, maternal sleep quality, and infant sleep quality, thereby providing empirical evidence and clinical reference for future maternal-infant health strategies.

## Literature review

Postpartum women face substantial physiological and psychological adjustments following childbirth, and comprehensive postpartum care plays a critical role in ensuring maternal and infant health. In Taiwan, the traditional postpartum practice known as “doing the month” originates from Chinese cultural customs and emphasizes dietary regulation, adequate rest, and minimized outings to promote recovery and prevent future health issues^4,11^. Postpartum care encompasses both maternal and infant care. According to the American College of Obstetricians and Gynecologists (ACOG), postpartum care should be regarded as an ongoing process rather than a one-time event to optimize health outcomes for mothers and infants^12^.

Postpartum women undergo key physiological processes including uterine involution, lochia discharge, and wound healing. Regardless of whether the delivery was vaginal or cesarean, proper wound care and infection prevention are essential^13^. Breast care during the lactation period is also vital; appropriate management helps prevent mastitis and nipple fissures, thereby supporting successful breastfeeding. Pelvic floor muscle rehabilitation and moderate postpartum exercise can strengthen core muscles and reduce the risk of pelvic relaxation or urinary incontinence^14,15^. Nutritional supplementation and regular routines are also essential for recovery. A balanced diet aids in wound healing, enhances breast milk quality, and restores energy. Adequate sleep helps alleviate fatigue and improve immunity. However, due to newborns’ irregular sleep patterns, mothers must adjust their own sleep schedules. Without sufficient rest, they risk impaired recovery and heightened risk of postpartum psychological conditions^3,16,17^.

Infant sleep behavior directly influences maternal circadian rhythms. A mother’s sleep quality often becomes governed by her infant’s needs rather than a regular sleep-wake cycle^2,18^. Studies indicate that mothers who rest when their infants sleep and receive sufficient family support to maintain at least 6 to 7 h of continuous sleep demonstrate significantly improved recovery outcomes^3^. Additionally, mothers should be equipped with basic newborn care skills including umbilical cord care, bathing, diaper changing, and feeding techniques. They must also be able to plan the infant’s nutritional intake and sleep schedule, as well as respond appropriately to common emergencies such as choking, regurgitation, aspiration, and abnormal body temperature^19–21^.

In recent years, with the increasing prevalence of digital technologies, nursing education has gradually shifted from traditional in-person instruction to hybrid and remote learning models^22^. Telecare, a modern integration of technology and healthcare management, provides real-time professional guidance through video calls, instant messaging, and interactive modules^22^. Research has shown that telecare significantly improves maternal self-care ability, increases maternal confidence, and reduces anxiety resulting from lack of knowledge^9,10^. Additionally, digital platforms offer continuous educational resources that enhance mothers’ caregiving abilities and parenting confidence^23,24^.

In summary, comprehensive postpartum care should address both physical and psychological needs while incorporating telecare technologies. These combined support systems can provide mothers with real-time and sustained health guidance, reduce postpartum stress, enhance maternal self-efficacy, and promote maternal-infant well-being. Such integrated care not only assists mothers in adapting to newborn caregiving responsibilities but also ensures optimal physical recovery and psychological adjustment.

## Methods

### Study design

This quasi-experimental study utilized a one-group repeated-measures design consisting of one baseline assessment and two follow-up assessments (post 1 and post 2) to evaluate the effects of the maternal–infant telecare program on postpartum maternal confidence, maternal sleep quality, and infant sleep quality among postpartum women. 

### Participants

Convenience sampling was used to recruit postpartum women from a postpartum nursing care center in Northern Taiwan. Participants who were able to communicate effectively, understand the study’s purpose, and voluntarily agree to participate were included if they met the following criteria: (1) stayed in the postpartum nursing care center for at least 30 days; (2) delivered a newborn with a gestational age between 37 and 42 weeks and a birth weight greater than 2500 g; (3) primarily cared for their infants for more than 10 h daily after discharge from the center; and (4) were able to communicate in Mandarin or Taiwanese and willing to provide informed consent. Participants were excluded if they met any of the following conditions: (1) postpartum women diagnosed with psychiatric disorders, depression, or pregnancy complications before or after delivery; and (2) infants diagnosed with hereditary or congenital conditions, such as congenital heart disease or cleft lips.

The G * power 3.1 program was used to calculate the number of participants required for the study. Based on a previous study^25^, the effect size was set to 0.4. Also, we assigned a significance level of 0.05 and a power of 0.95 to calculate the sample size using a single group means comparison; the result was 70. We tried to recruit 84 participants considering that the dropout rate is 20%, but only 82 participants were recruited.

### Intervention program

The maternal-infant telecare program is a structured six-week intervention aimed at enhancing postpartum maternal caregiving confidence and infant care knowledge following discharge from postpartum nursing care center. Upon discharge, participants are enrolled in the Smart Care Platform, named “infancixhome”, a digital platform that provides tailored caregiving content and real-time consultation services. The intervention comprises three primary components: maternal and infant care modules, video consultation, and messaging support.

### Components of the maternal-infant telecare program

#### Maternal and infant care videos with instruction leaflets

The maternal and infant care modules are designed to address critical aspects of maternal and infant care through a structured, evidence-based approach. These materials consist of two main components—educational videos and instruction leaflets, which are systematically designed to provide guidance in the following areas:

- Maternal recovery and infant health care.
- Nutritional care.
- Safe sleep practices and routines for infant.
- 3Q Development: Physical Quotient (PQ), Intelligence Quotient (IQ), and Emotional Quotient (EQ).
- Safe and healthy living environment.

A total of 45 educational videos and instruction leaflets are automatically distributed daily through the infancixhome. The videos deliver step-by-step demonstrations and practical caregiving strategies, while the instruction leaflets provide concise, easy-to-follow written guidelines that mirror the video content. This synchronized distribution model ensures that postpartum women receive structured, timely information that reinforces learning and promotes practical application in daily care routines.

#### Video consultation

The Video Consultation service is structured to provide consistent professional guidance over the six-week intervention. Consultations are conducted once weekly for 30 min, resulting in a total of six sessions. To ensure continuity and personalized care, the same online nurse facilitates all six sessions. This approach allows the nurse to monitor maternal progress, address emerging caregiving challenges, and provide tailored health guidance effectively. During these sessions, postpartum women receive:

- Customized health education.
- Evidence-based recommendations for infant care.
- Real-time solutions to specific caregiving concerns.

The structured interaction enhances maternal confidence and caregiving competence, promoting effective maternal-infant bonding and improved health outcomes.

#### Messaging support

The messaging support service is available on the infancixhome from Monday to Friday, 09:00 to 18:00 throughout the entire intervention period. This service is specifically designed to address real-time caregiving concerns encountered by postpartum women.

### Instruments

#### Demographic questionnaire (DQ)

The DQ requested participants’ age, educational level, marital status, total household income, parity, parturition method, sex of this infant, method of this infant’s feeding, and post-discharge infant caregiving assistance.

#### Maternal confidence questionnaire (MCQ)

This study adopted a self-developed Maternal Confidence Questionnaire (MCQ) to evaluate mothers’ confidence in their parenting skills and their ability to understand their infants’ needs. The content of the scale was developed with reference to relevant studies^26–28^ and consists of 14 items, including 12 positively worded and 2 negatively worded statements. Each item is rated on a 5-point Likert scale (1 = Never, 2 = Rarely, 3 = Sometimes, 4 = Often, 5 = Always), with total scores ranging from 14 to 70; higher scores indicate greater maternal confidence. Content validity was evaluated by a panel of five experts, including two doctoral-level nursing faculty members and three senior clinical nursing specialists, who systematically reviewed each item for relevance to the scale’s objectives. Using a 5-point rating scale (1 = Highly inappropriate to 5 = Highly appropriate), the experts assessed the appropriateness of each item. Based on their feedback, wording and content were refined. The item-level content validity index (I-CVI) reached 0.80, and the scale-level content validity index (S-CVI) reached 0.87, indicating satisfactory content validity. In the present study, the MCQ demonstrated good internal consistency, with a Cronbach’s α coefficient of 0.88.

#### Postpartum sleep quality scale (PSQS)

The PSQS developed by Chen (2011), was utilized to assess the sleep quality of postpartum women over the preceding two weeks^29^. The scale comprises 14 items, each designed to evaluate specific aspects of maternal sleep quality. Responses are recorded on a 5-point Likert scale (0 = never, 1 = few, 2 = sometimes, 3 = often, 4 = almost always. Lower total scores represent better sleep quality, with the overall score ranging from 0 to 56. This scale demonstrated good internal consistency, with a Cronbach’s alpha coefficient of 0.81.

#### Brief infant sleep questionnaire (BISQ)

Chang (2014) adapted the BISQ^30^, originally developed by Dr. Avi Sadeh in 2004, into a Chinese version and evaluated its reliability and validity. The Chinese version of the BISQ consists of various items designed to measure key aspects of infant sleep, including nighttime sleep duration, daytime nap duration, sleep onset latency, frequency of nighttime awakenings, and the time required to soothe the infant back to sleep. The BISQ Chinese version demonstrated acceptable criterion-related validity and reliability, with Cronbach’s α coefficients ranging from 0.61 to 0.63.

### Data collection

The intervention and data collection for this study were conducted from October 2022 to January 2023. Firstly, to recruit the participants, we contacted a postpartum nursing care center in Northern Taiwan, explained the study’s purpose, and requested their cooperation. Subsequently, data collection and intervention were conducted. In addition, the effectiveness of the intervention was evaluated at three time points: baseline, six weeks, and twelve weeks post-intervention.

### Ethical considerations

This study was reviewed and approved by the Institutional Review Board (IRB) of Show Chwan Memorial Hospital through a full board review process (Approval No. 1110102) on January 12, 2022. Prior to the commencement of the study, the research objectives and protocols were detailed to all participants, along with comprehensive information regarding the protection of human rights and the guarantee of data anonymity. Consequently, all participants were fully apprised of the study’s aims, procedures, and their respective rights, and written informed consent was obtained from each individual before enrollment. Furthermore, participants were explicitly informed of their voluntary right to decline participation or withdraw from the study at any stage without any adverse impact on the quality of clinical care received.

### Data analysis

All statistical analyses were performed using the SPSS 24 software (IBM Corp, Armonk, NY, USA). First, participants’ demographics were examined using descriptive statistics to provide an overview of the sample characteristics. Second, generalized estimating equations (GEE) were used to examine changes in maternal confidence and sleep quality among postpartum women, as well as infants’ sleep quality. This approach has been suggested as an effective method for analyzing longitudinal data from the same subjects measured at different time points.

## Results

### Demographic characteristics

This study included 82 postpartum women. The demographic characteristics of the participants are summarized in Table 1. The average age was 34.89 years (*SD* = 4.34). Most participants held a college degree (*n* = 50, 61.0%) and were married (*n* = 78, 95.1%). Most of the postpartum women held the role of housewives, accounting for 61 participants (74.39%). Nearly half reported a monthly household income of NT 100,000 or more (*n* = 38, 46.3%), and 75 participants (91.5%) were primiparas. Regarding the delivery method, 44 participants (53.7%) underwent a cesarean section. Slightly more than half of the infants were boys (*n* = 43, 52.4%), and the predominant feeding method was mixed feeding (*n* = 48, 58.5%). Post-discharge infant caregiving was primarily supported by husbands (*n* = 48, 58.5%) or managed independently by the postpartum women (*n* = 25, 30.5%). Two participants dropped out of the study due to work-related commitments.

**Table 1:** Demographic characteristics (*N* = 82).

Variable	Mean ± SD	*n* (%)
Age	34.89 ± 4.34	
Educational level		
Less than junior high school	1	(1.2%)
High school	3	(3.7%)
College degree	50	(61.0%)
Master degree	28	(34.1%)
Marital status		
Unmarried	3	(3.7%)
Married	78	(95.1%)
Divorce	1	(1.2%)
Women’s roles		
Housewives	61	(74.39%)
Career women	21	(25.61%)
Total household income (per month)		
< NT 70,000	15	(18.3%)
NT 70,000-100,000	29	(35.4%)
≥ NT 100,000	38	(46.3%)
Parity		
Primiparas	75	(91.5%)
Multiparas	7	(8.5%)
Parturition method		
Labor	38	(46.3%)
Cesarean section	44	(53.7%)
Sex of infant		
Girl	39	(47.6%)
Boy	43	(52.4%)
Method of infant’s feeding		
Formula	20	(24.4%)
Breast	14	(17.1%)
Mixed	48	(58.5%)
Post-discharge infant caregiving assistance		
Husband	48	(58.5%)
All myself	25	(30.5%)
Nanny	3	(3.6%)
Mother-in-law	2	(2.4%)
Mother	4	(4.9%)

*SD:* standard deviation. Percentages may not sum to 100% due to rounding.

#### Postpartum maternal confidence

The intervention’s impact on postpartum maternal confidence was assessed at three time points: baseline, six weeks, and twelve weeks post-intervention. The maternal confidence was higher than pre-intervention and post-intervention (baseline: 43.99, post 1: 49.20), and further increased at post 2 (51.72); the result was statistically significant (*χ*^*2*^ = 174.73, *p* < 0.001, Cohen’s *d* = 0.51), indicating a substantial enhancement in maternal confidence over the intervention period. The effect size, represented by Cohen’s *d*, was 0.51, suggesting a moderate impact of the intervention on boosting postpartum women’s confidence in caregiving practices. This improvement highlights the effectiveness of the program in empowering postpartum women with the necessary skills and confidence to care for their infants during the early postpartum phase.

#### Postpartum sleep quality

The postpartum sleep quality scores decreased significantly from 23.35 (baseline) to 20.85 (post 1) and sustained a significantly lower score of 19.33 at post 2, indicating improved sleep quality. The GEE analysis confirmed that the differences across time points were statistically significant (χ^2^ = 31.43, *p* < 0.001, Cohen’s *d* = -0.24). These findings suggest that the intervention effectively enhanced sleep quality for postpartum women (Table 2).

**Table 2:** Effects of a maternal-infant telecare program on postpartum maternal confidence and sleep quality (*N* = 82).

Variables	Baseline	Post 1 (6 week)	Post 2 (12 week)	X^2^	*p* value	Cohen’s d
	Mean ± SE	Mean ± SE	Mean ± SE			
MCQ	43.99 ± 0.85	49.20 ± 0.73	51.72 ± 0.68	174.73	< 0.001**	0.51
PSQS	23.35 ± 0.94	20.85 ± 0.87	19.33 ± 0.90	31.43	< 0.001**	-0.24

SE: standard error.

#### Infants’ sleep quality

The intervention significantly improved infants’ sleep quality. Total sleep duration increased significantly from 473.41 min (baseline) to 499.02 min (post 1) and maintained a significantly elevated score of 540.00 min at post 2 (*χ*^*2*^ = 24.66, *p* < 0.001, Cohen’s *d* = 0.33). Daytime sleep notably decreased from 434.63 min (baseline) to 283.90 min (post 2), indicating a shift towards more consolidated nighttime rest (*χ*^*2*^ = 118.72, *p* < 0.001, Cohen’s *d* = -0.59). Nighttime awakenings were also reduced significantly from 2.77 (baseline) to 1.63 (post 1) and further to 1.12 (post 2) (*χ*^*2*^ = 68.28, *p* < 0.001, Cohen’s *d* = -0.53). Nocturnal wakefulness time was also reduced, from 90.98 min (baseline) to 47.93 (post 1) and then to 19.84 min (post 2) (χ^2^ = 117.42, *p* < 0.001, d = -0.54), reflecting improved sleep continuity. These results, summarized in Table 3, highlight the effectiveness of the maternal-infant telecare program in optimizing infant sleep patterns.

**Table 3:** Effects of a maternal-infant telecare program on infants’ sleep quality (*N* = 82).

Variables	Baseline	Post 1 (6 week)	Post 2 (12 week)	X^2^	*p* value	Cohen’s d
	Mean ± SE	Mean ± SE	Mean ± SE			
Nocturnal sleep duration (mins)	473.41 ± 12.16	499.02 ± 11.14	540.00 ± 8.72	24.66	< 0.001**	0.33
Daytime sleep duration (mins)	434.63 ± 11.82	382.44 ± 13.80	283.90 ± 11.29	118.72	< 0.001**	-0.59
Night wakings, number (no.)	2.77 ± 0.19	1.63 ± 0.14	1.12 ± 0.11	68.28	< 0.001**	-0.53
Nocturnal wakefulness (mins)	90.98 ± 7.85	62.99 ± 7.50	19.84 ± 2.66	117.42	< 0.001**	-0.54

SE: standard error.

## Discussion

The demographic characteristics of the participants provide important insights into the effectiveness of the maternal-infant telecare program. Most participants were well-educated (95.1% held a college degree or higher), married (95.1%), and primarily housewives (74.39%), with 91.5% being first-time mothers. These factors are known to influence postpartum adaptation and caregiving confidence^7,19^. Additionally, more than half of the mothers underwent cesarean deliveries (53.7%), highlighting the need for structured support during recovery and infant care^31^. After discharge, infant caregiving was mainly managed by mothers, with support from their husbands (58.5%). This demonstrates the importance of family involvement in newborn care. Prior studies suggest that spousal support helps reduce maternal stress and enhances confidence in caregiving^32,33^. The inclusion of telecare in this study provided continuous guidance, which may have further strengthened maternal caregiving abilities.

The findings of this study indicate that the maternal-infant telecare program significantly enhanced postpartum maternal confidence over the intervention period. Maternal confidence scores increased from 43.99 at baseline to 49.20 at six weeks and further improved to 51.72 at twelve weeks. This substantial improvement, as demonstrated by a statistically significant change (χ^2^ = 174.73, *p* < 0.001) and a moderate effect size (Cohen’s d = 0.51), suggests that structured telecare support can effectively bolster maternal confidence during the early postpartum period. This result aligns with previous studies emphasizing the positive impact of telecare interventions on maternal confidence and caregiving capabilities. For example, Kongsaenkaew et al. (2024) found that postnatal telehealth support programs significantly improved maternal self-efficacy and caregiving confidence by providing timely guidance and emotional support^34^. Similarly, a study by Javadi et al. (2024) demonstrated that continuous telecare follow-up significantly enhanced maternal self-assurance in handling infant care tasks during the critical early postpartum period^9^. These findings support the notion that ongoing telecare support can mitigate the challenges faced by postpartum women, particularly during the transition from structured care in postpartum nursing care centers to independent caregiving at home.

Furthermore, the enhancement in maternal confidence observed in this study may be attributed to the regular access to health information and real-time consultation provided by the telecare platform. The consistent improvement observed from six weeks to twelve weeks indicates that prolonged exposure to telecare services may have a cumulative effect, enhancing maternal confidence over time. In conclusion, the maternal-infant telecare program effectively enhances postpartum maternal confidence by providing structured guidance and emotional support during the critical postpartum period. This program not only addresses the immediate concerns of mothers, especially those facing the challenges of postpartum care, but also enhances their confidence in caregiving, suggesting its potential as a scalable intervention in postpartum care settings.

The present study observed changes in parent-reported infant sleep patterns across the postpartum period among mothers who participated in a maternal–infant telecare program. Specifically, the parent-reported total sleep duration significantly increased from 473.41 min at baseline to 499.02 min at Post 1 and further to 540.00 min at Post 2 (χ^2^ = 24.66, *p* < 0.001, Cohen’s *d* = 0.33). These findings reflect maternal perceptions of longer infant sleep duration over time rather than objective changes in infant sleep physiology. Within the context of this study, such perceptions are clinically meaningful, as maternal interpretations of infant sleep have been shown to be associated with caregiving confidence, stress, and daily caregiving decision-making^35–37^.

In addition to total sleep duration, changes were also observed in the distribution of infant sleep. Parent-reported daytime sleep duration decreased from 434.63 min at baseline to 283.90 min at Post 2 (χ^2^ = 118.72, *p* < 0.001, Cohen’s d = − 0.59), suggesting a perceived shift toward more consolidated nighttime rest. This pattern is consistent with well-documented developmental trajectories of infant sleep, in which nocturnal sleep consolidation increases and daytime sleep gradually decreases during the first months of life^38^. Accordingly, these changes should be interpreted primarily within the framework of normal biological maturation. Reductions in parent-reported nighttime awakenings were also observed, decreasing from 2.77 at baseline to 1.63 at Post 1 and further to 1.12 at Post 2 (χ^2^ = 68.28, *p* < 0.001, Cohen’s d = − 0.53). Rather than indicating a direct intervention effect on infant sleep regulation, these findings likely reflect changes in maternal perceptions of sleep continuity and infant regulation during the postpartum period. Previous research has highlighted that parental support and guidance delivered through telehealth may shape how caregivers interpret and respond to infant sleep behaviors, thereby influencing perceived sleep stability^39,40^. Importantly, infant sleep development during early infancy is strongly influenced by biological maturation, and improvements in sleep duration and consolidation would be expected to occur over time regardless of intervention exposure^18^. In the present study, the telecare program should therefore be understood as operating within this developmental context, potentially supporting mothers in recognizing sleep cues, establishing caregiving routines, and interpreting infant sleep patterns with greater confidence, rather than as a mechanism that alters the underlying trajectory of infant sleep maturation.

Beyond the observed improvements in maternal and infant outcomes, the underlying mechanisms of the telecare program’s effectiveness can be understood through Bandura’s Social Cognitive Theory. The program enhanced maternal self-efficacy by combining mastery experiences (daily caregiving), vicarious experiences (educational videos), and verbal persuasion (video consultations). These aligned with known pathways to behavior change and psychological empowerment. Furthermore, the reduction in sleep disturbances among mothers may reflect decreased anxiety and improved coping, as confidence in caregiving has been shown to mediate maternal sleep quality^16^. These findings suggest that telecare programs not only fill a service gap post-discharge but also catalyze psychological resilience and adaptive caregiving behaviors through established behavioral frameworks.

In summary, this study observed parent-reported changes in infant sleep patterns across the postpartum period among mothers participating in a maternal–infant telecare program. These changes, including longer perceived sleep duration and fewer reported nighttime awakenings, occurred alongside improvements in maternal confidence and maternal sleep quality. Importantly, the infant sleep findings should be interpreted as perception-based outcomes that are temporally associated with participation in the telecare program and concurrent normal infant sleep maturation, rather than as evidence of a direct intervention effect on infant sleep physiology. Together, these findings highlight the potential role of telecare programs in supporting maternal caregiving confidence and shaping maternal perceptions of infant regulation during the transition to home-based postpartum care.

### Strengths and limitations

This study examined the implementation of a maternal–infant telecare program during the transition from postpartum nursing care centers to home-based care and observed positive changes in maternal confidence, maternal sleep quality, and parent-reported infant night waking patterns during the postpartum period. The real-world implementation of the program supports its feasibility and potential applicability in routine maternal care settings. However, the study’s cross-sectional design, convenience sampling, and small sample size attributable to the limited number of institutions providing maternal–infant telecare restrict its ability to establish causality and generalize the findings. Data were collected from a single postpartum nursing care center, and self-reported measures may introduce bias. In particular, infant sleep outcomes were based on parent-reported measures and should be interpreted as reflecting maternal perceptions rather than objective assessments of infant sleep physiology. In addition, the BISQ demonstrated relatively low internal consistency in this study, which may further limit the reliability of the infant sleep measurements. Additionally, the absence of a control group limits comparisons with standard care. Future studies should consider longitudinal designs, broader sampling across multiple institutions, and the incorporation of objective measures to further evaluate the program’s long-term impact and generalizability.

### Implications and sustainability

Given the program’s favorable outcomes and its delivery via an existing digital platform, there is strong potential for scalability within Taiwan’s maternal-child health system. The program’s asynchronous components reduce reliance on clinical staff, while scheduled consultations ensure personalization and accountability. These features enhance sustainability and make the intervention adaptable across diverse healthcare settings. Moreover, the use of culturally aligned educational content (e.g., postpartum nutrition, traditional rest practices) ensures relevance and acceptability among target users. Future iterations of the program could incorporate machine learning–driven personalization or integration with electronic health records to further optimize care delivery.

## Conclusion

The findings of this study demonstrate that the maternal-infant telecare program effectively improves postpartum maternal confidence, enhances sleep quality for postpartum women, and reduces infants’ night waking. This program extends support beyond postpartum nursing care centers, helping postpartum women transition to home care with greater confidence and clearer guidance in caring for themselves and their babies. The maternal-infant telecare program serves as a valuable solution for bridging the gap between clinical care and home-based care, empowering postpartum women with practical skills and confidence. Future research should explore its long-term effects on maternal well-being, infant development, and family health. Overall, the maternal-infant telecare program is a promising innovation in postpartum care that merits wider adoption to support postpartum women and infants during the transition from care centers to home.

## Data Availability

Data available on request due to restrictions regarding privacy, legal and ethical concerns. The data presented in this study are available on request from the corresponding author.

## References

[1] Lefèvre, M. et al. Impact of shortened length of stay for delivery on the required bed capacity in maternity services: Results from forecast analysis on administrative data. *BMC Health Serv. Res.* **19**, 637. 10.1186/s12913-019-4500-8 (2019). 31488147 10.1186/s12913-019-4500-8 PMC6729074

[2] Schwartz, E. & Villa, J. K. Navigating the Postpartum Period: Hormonal Changes and Essential Care for Women. (2025). 10.5772/intechopen.1008428.

[3] Benedetto, L., Peña, F., Rivas, M., Ferreira, A. & Torterolo, P. The integration of the maternal care with sleep during the postpartum period. *Sleep Med. Clin.* **18** (4), 499–509. 10.1016/j.jsmc.2023.06.013 (2023). 38501522 10.1016/j.jsmc.2023.06.013

[4] Ding, G. et al. Doing the month and postpartum depression among Chinese women: A Shanghai prospective cohort study. *Women Birth*. **33** (2), e151–e158. 10.1016/j.wombi.2019.04.004 (2020). 31060983 10.1016/j.wombi.2019.04.004

[5] Keyser-Verreault, A. Reinventing chinese postnatal rituals: Doing the month in taiwanese postpartum nursing centres. *Anthropol. Forum*. **34** (4), 459–479. 10.1080/00664677.2024.2444443 (2024).

[6] Martianto, Y. H., Indradewa, R., Kustiawan, U. & Pamungkas, R. A. Operational planning of’ remedies’ postpartum care center: Ensuring of service providing to consumers. *J. Manage. Economic Financial*. **3** (3), 1–18. 10.59261/jmef.v3i3.80 (2025).

[7] Kristensen, I. H., Simonsen, M., Trillingsgaard, T., Pontoppidan, M. & Kronborg, H. First-time mothers’ confidence, mood and stress in the first months postpartum: A cohort study. *Sex. Reproductive Healthc.* **17**, 43–49. 10.1016/j.srhc.2018.06.003 (2018). 10.1016/j.srhc.2018.06.003 30193719

[8] Schobinger, E., Vanetti, M., Ramelet, A. S. & Horsch, A. Social support needs of first-time parents in the early-postpartum period: A qualitative study. *Front. Psychiatry*. **13**, 1043990. 10.3389/fpsyt.2022.1043990 (2022). 36590631 10.3389/fpsyt.2022.1043990 PMC9794858

[9] Javadi, M., Ebrahimi, S. & Hosseini, M. The effect of tele-continuous midwifery care on maternal functioning and neonatal perception among Iranian primiparous mothers: A randomized field trial. *J. Clin. Med.* **13** (20), 6062. 10.3390/jcm13206062 (2024). 39458012 10.3390/jcm13206062 PMC11508779

[10] Kikuchi, K. et al. Nakashima, N. Telehealth care for mothers and infants to improve the continuum of care: Protocol for a quasi-experimental study. *JMIR Res. Protocols*. **11** (12), e41586. 10.2196/41586 (2022). 10.2196/41586 PMC9801263 36520523

[11] Wong, J. & Fisher, J. The role of traditional confinement practices in determining postpartum depression in women in Chinese cultures: a systematic review of the English language evidence. *J. Affect. Disord.* **116** (3), 161–169. 10.1016/j.jad.2008.11.002 (2009). 19135261 10.1016/j.jad.2008.11.002

[12] McKinney, J., Keyser, L., Clinton, S. & Pagliano, C. ACOG committee opinion 736: Optimizing postpartum care. *Obstet. Gynecol.* **132** (3), 784–785. 10.1097/AOG.0000000000002849 (2018). 30134408 10.1097/AOG.0000000000002849

[13] Lewin, S. et al. Wound healing after vaginal delivery, episiotomy, and cesarean section delivery among women with IBD: Results from the piano registry. *Inflamm. Bowel Dis.* izae310. 10.1093/ibd/izae310 (2025). 10.1093/ibd/izae310 39779464

[14] Chen, S., Wang, L., Guo, H., Jiang, M. & Wang, X. Comparative analysis of pelvic floor muscle damage postpartum: Vaginal delivery vs. cesarean section. *Int. J. Clin. Pract.* **2024** (1), 1169924. 10.1155/2024/1169924 (2024).

[15] Frawley, H., Shelly, B., Morin, M., Bernard, S., Bø, K., Digesu, G. A., & Voelkl Guevara,J. An International Continence Society (ICS) report on the terminology for pelvic floor muscle assessment. *Neurourol. Urodyn.* **40**(5), 1217–1260. 10.1002/nau.24658 (2021). 10.1002/nau.24658 33844342

[16] Mazandarani, A. A. & Zare Bahramadbadi, M. Maternal Self-Efficacy as a Moderator in the Relationship between Infant/Toddler Sleep and Maternal Mental Health. *Behav. sleep. Med.* 1–11. 10.1080/15402002.2025.2483961 (2025). 10.1080/15402002.2025.2483961 40125868

[17] Ran-Peled, D., Horwitz, A., Finkelstein, O., Atzaba-Poria, N. & Tikotzky, L. A longitudinal study of postpartum maternal sleep and sensitivity: Examining depressive symptoms and social support as moderators. *J. Sleep Res.* **e70056**10.1111/jsr.70056 (2025). 10.1111/jsr.70056 PMC12426717 40170203

[18] de Groot, E. R., Dudink, J. & Austin, T. Sleep as a driver of pre-and postnatal brain development. *Pediatr. Res.* **96** (6), 1503–1509. 10.1038/s41390-024-03371-5 (2024). 38956219 10.1038/s41390-024-03371-5 PMC11624135

[19] Guo, K., Shang, X. & Deng, X. The effects of a newborn care education program on mothers’ self-confidence, care knowledge, and breastfeeding behavior: A systematic review and meta‐analysis. *Public Health Nurs.* **42** (1), 395–410. 10.1111/phn.13484 (2025). 39517114 10.1111/phn.13484

[20] Furtak, S. L., Gay, C. L., Kriz, R. M., Bisgaard, R., Bolick, S. C., Lothe, B., & Franck, L. S. What parents want to know about caring for their preterm infant: A longitudinal descriptive study. *Patient Educ. Counseling* **104** (11), 2732–2739. 10.1016/j.pec.2021.04.011 (2021). 10.1016/j.pec.2021.04.011 33966954

[21] National Health Service. Helping your baby to sleep. (2025). https://www.nhs.uk/baby/caring-for-a-newborn/helping-your-baby-to-sleep/.

[22] Johnsen, H. M., Briseid, H. S., Brodtkorb, K., Slettebø, Å. & Fossum, M. Nursing students’ perceptions of combining hands-on simulation with simulated patients and a serious game in preparing for clinical placement in home healthcare: A qualitative study. *Nurse Educ. Today*. **97**, 104675. 10.1016/j.nedt.2020.104675 (2021). 33302184 10.1016/j.nedt.2020.104675

[23] Chua, C. M. S., Mathews, J., Ong, M. S. B., Liew, K. K. & Shorey, S. Use of telelactation interventions to improve breastfeeding outcomes among mothers: A mixed-studies systematic review. *Women Birth*. **36** (3), 247–256. 10.1016/j.wombi.2022.06.011 (2023). 35792035 10.1016/j.wombi.2022.06.011

[24] Solís-Cordero, K., Duarte, L. S. & Fujimori, E. Effectiveness of remotely delivered parenting programs on caregiver-child interaction and child development: A systematic review. *J. Child Fam. stud.* **31** (11), 3026–3036. 10.1007/s10826-022-02328-8 (2022). 35615461 10.1007/s10826-022-02328-8 PMC9123827

[25] Kang, H. Sample size determination and power analysis using the G* Power software. *J. Educ. Eval. Health Professions*. **18**10.3352/jeehp.2021.18.17 (2021). 10.3352/jeehp.2021.18.17 PMC8441096 34325496

[26] Badr, L. K. Further psychometric testing and use of the maternal confidence questionnaire. *Issues Compr. Pediatr. Nurs.* **28** (3), 163–174. 10.1080/01460860500227572 (2005). 16251162 10.1080/01460860500227572

[27] Liu, C. C. & Chen, Y. C. Concept analysis of maternal confidence. *Chang. Gung Nurs.* **22** (4), 472–478. 10.6386/CGN.201112_22(4 (2011).

[28] Kuo, S. F., Chen, Y. C., Mao, H. C. & Tsou, K. I. Effects of individual nursing instruction on infant care knowledge and maternal confidence of primiparas. *Nurs. Res.* **8** (2), 152–164. 10.7081/NR.200004.0152 (2000).

[29] Chan, C. H. & Development of Postpartum Sleep Quality Scale and the Effects of Walking Exercise and Herbal Tea on Postpartum Sleep Quality. National Science and Technology Council, Taipei City, Taiwan. Available online: (2011). https://www.grb.gov.tw/search/planDetail?id=2199245 (accessed on 02 August 2025).

[30] Chang, Y. T. A. & Preliminary Study of Reliability and Validity of the Chinese Version Brief Infant Sleep Questionnaire. Master’s Thesis, Chang Gung University, Taoyuan City, Taiwan. Available online: (2014). https://hdl.handle.net/11296/e4ja63 (accessed on 02 August 2025).

[31] American College of Obstetricians and Gynecologists. Optimizing Postpartum Care. (2021). Retrieved from https://www.acog.org/clinical/clinical-guidance/committee-opinion/articles/2018/05/optimizing-postpartum-care.

[32] Devandra, M. & Nandita, I. D. Spousal Support and Postpartum Depression: Impact of Partner Involvement on Maternal Mental Health. *Acta Psychologia*. **2** (2), 58–65 (2023). https://psychologia.pelnus.ac.id/index.php/Psychologia/article/view/37

[33] Topaloğlu Ören, E. D. & Cengiz, B. The spousal support and breastfeeding self-efficacy between mothers of healthy babies and babies in the NICU: A comparative and correlational study. *Women Health*. 1–14. 10.1080/03630242.2025.2498522 (2025). 10.1080/03630242.2025.2498522 40320734

[34] Kongsaenkaew, K., Rungamornarat, S. & Payakkaraung, S. Enhancing maternal self-efficacy in caring for preterm infants with ventilator through a telehealth program: a randomized controlled trial. *Pac. Rim Int. J. Nurs. Res.* **28** (1), 88–102. 10.60099/prijnr.2024.264464 (2024).

[35] Cook, F. et al. Profiles and predictors of infant sleep problems across the first year. *J. Dev. Behav. Pediatr.* **41** (2), 104–116. 10.1097/DBP.0000000000000733 (2020). 31567724 10.1097/DBP.0000000000000733

[36] Vertsberger, D., Tikotzky, L., Baruchi, O. & Knafo-Noam, A. Parents’ perceptions of infants’ nighttime sleep patterns predict mothers’ negativity: A longitudinal study. *J. Dev. Behav. Pediatr.* **42** (4), 307–313. 10.1097/dbp.0000000000000899 (2020). 10.1097/DBP.0000000000000899 33337599

[37] Zanetti, N., D’Souza, L., Tchernegovski, P. & Blunden, S. Parents’ perceptions of the quality of infant sleep behaviours and practices: A qualitative systematic review. *Infant Child. Dev.* **32** (1), e2369. 10.1002/icd.2369 (2023).

[38] Henderson, J. M., Blampied, N. M. & France, K. G. Longitudinal study of infant sleep development: Early predictors of sleep regulation across the first year. *Nat. Sci. Sleep.* 949–957. 10.2147/NSS.S240075 (2020). 10.2147/NSS.S240075 PMC7667498 33204198

[39] Shen, X., Huang, P., Liu, Q., Guo, Y. & Zheng, L. Telehealth based parental support over 6 months improves physical activity and sleep quality in children with autism: a randomized controlled trial. *Front. Pead.* **12**, 1496827. 10.3389/fped.2024.1496827 (2024). 10.3389/fped.2024.1496827 PMC11543462 39525834

[40] Traynor, N. M. et al. Supporting families with complex early parenting needs through a virtual residential parenting service: an investigation of outcomes, facilitators and barriers. *J. Clin. Nurs.* **33** (3), 1122–1133. 10.1111/jocn.16927 (2024). 37962242 10.1111/jocn.16927

